# Infection and upregulation of proinflammatory cytokines in human brain vascular pericytes by human cytomegalovirus

**DOI:** 10.1186/1742-2094-9-95

**Published:** 2012-05-18

**Authors:** Donald J Alcendor, Ashley M Charest, Wen Qin Zhu, Hollie E Vigil, Susan M Knobel

**Affiliations:** 1 Department of Microbiology and Immunology, Center for AIDS Health Disparities Research, Meharry Medical College, School of Medicine, 1005 Dr. D.B. Todd Jr. Blvd, Nashville, TN, 37208-3599, USA

**Keywords:** Cytomegalovirus, pericytes, astrocytes, BMVECs, neuroinflammation, blood–brain barrier, virus replication, cytokines

## Abstract

**Background:**

Congenital human cytomegalovirus (HCMV) infections can result in CNS abnormalities in newborn babies including vision loss, mental retardation, motor deficits, seizures, and hearing loss. Brain pericytes play an essential role in the development and function of the blood–brain barrier yet their unique role in HCMV dissemination and neuropathlogy has not been reported.

**Methods:**

Primary human brain vascular pericytes were exposed to a primary clinical isolate of HCMV designated ‘SBCMV’. Infectivity was analyzed by microscopy, immunofluorescence, Western blot, and qRT-PCR. Microarrays were performed to identify proinflammatory cytokines upregulated after SBCMV exposure, and the results validated by real-time quantitative polymerase chain reaction (qPCR) methodology. *In situ* cytokine expression of pericytes after exposure to HCMV was examined by ELISA and *in vivo* evidence of HCMV infection of brain pericytes was shown by dual-labeled immunohistochemistry.

**Results:**

HCMV-infected human brain vascular pericytes as evidenced by several markers. Using a clinical isolate of HCMV (SBCMV), microscopy of infected pericytes showed virion production and typical cytomegalic cytopathology. This finding was confirmed by the expression of major immediate early and late virion proteins and by the presence of HCMV mRNA. Brain pericytes were fully permissive for CMV lytic replication after 72 to 96 hours in culture compared to human astrocytes or human brain microvascular endothelial cells (BMVEC). However, temporal transcriptional expression of pp65 virion protein after SBCMV infection was lower than that seen with the HCMV Towne laboratory strain. Using RT-PCR and dual-labeled immunofluorescence, proinflammatory cytokines CXCL8/IL-8, CXCL11/ITAC, and CCL5/Rantes were upregulated in SBCMV-infected cells, as were tumor necrosis factor-alpha (TNF-alpha), interleukin-1 beta (IL-1beta), and interleukin-6 (IL-6). Pericytes exposed to SBCMV elicited higher levels of IL-6 compared to both mock-infected as well as heat-killed virus controls. A 6.6-fold induction of IL-6 and no induction TNF-alpha was observed in SBCMV-infected cell supernatants at 24 hours postinfection. Using archival brain tissue from a patient coinfected with HCMV and HIV, we also found evidence of HCMV infection of pericytes using dual-label immunohistochemistry, as monitored by NG2 proteoglycan staining.

**Conclusion:**

HCMV lytic infection of primary human brain pericytes suggests that pericytes contribute to both virus dissemination in the CNS as well as neuroinflammation.

## Background

Human cytomegalovirus (HCMV) is a ubiquitous pathogen and is the most common infectious cause of congenital disease [1-4]. Congenital infections caused by HCMV represent the leading infectious cause of mental retardation and deafness in children [5,6]. Other abnormalities in newborns include vision loss, motor deficits, seizures, and sensorineural hearing loss [7-9]. With only 10 to 15% of children presenting with symptomatic disease at birth, HCMV can also result in long-term progressive neuropathology in children who are asymptomatic at birth. It is estimated that approximately 8,000 children are affected each year in the United States with some form of neuropathology associated with a cytomegalovirus (CMV) congenital infection [7]. In addition, HCMV, a common opportunistic infection in patients with HIV/AIDS [10], is often found in brain tissue from these patients [11] where it can be associated with cytomegalovirus-induced encephalitis [12,13].

The blood–brain barrier (BBB) interfaces the peripheral circulation and the central nervous system (CNS) allowing nutrients into the CNS and preventing blood-borne pathogens from harming the brain. This barrier is an elaborate network of tight junctions (TJ) between capillary endothelial cells that lack fenestrae and have a reduced capacity for pinocytosis [14,15]. The TJ of the capillary endothelium is supported by astrocytic endfeet and pericytes. Cerebral vascular pericytes (CNS pericytes) have been shown to enhance TJ barrier function, stimulate expression of TJ proteins and reduce the paracellular permeability of the capillary endothelium [16-19]. Pericytes are adult multipotent, contractile and migratory stem cells [20,21] that surround capillaries and actively communicate with other cells of the neurovasculature, including endothelial cells, astrocytes and neurons. Completely surrounded by a basal lamina, they also contribute to the deposition of the basal lamina during vascular development and angiogenesis [22]. Although a critical cellular component for the development and function of the BBB, the role of pericytes in HCMV infection and dissemination has largely been ignored [23]. Rather, to date, astrocytes and brain microvascular endothelial cells (BMVEC) cells have been implicated as cell types that support HCMV dissemination at the blood–brain barrier level [7].

To our knowledge, no studies have reported on the interaction between human cytomegalovirus and brain vascular pericytes. However in 1990, Price *et al*. [24] demonstrated murine CMV (MCMV) infection of pericytes in brown and white adipose tissue of young adult infected mice. These mice later developed viral-induced inflammatory lesions in peripancreatic and salivary gland adipose tissues. In the present study, we investigated the infectivity of human brain vascular pericytes by HCMV using a primary isolate from a child with disseminated HCMV disease. Infection was determined by monitoring pericytes for HCMV cytopathology using standard biochemical assays for viral proteins and viral mRNA. Proinflammatory cytokines expressed in exposed pericytes were identified by microarrays and confirmed by dual-labeled immunofluorescence and real-time PCR (qRT-PCR).

This is the first report to date that investigates the infectivity of human brain pericytes for HCMV and their role in viral dissemination in the CNS. It supports the postulate that pericytes are more permissive for CMV lytic replication compared to BMVEC or astrocytes, and that pericytes could serve as amplification reservoirs for HCMV. Finally, pericyte exposure to HCMV induced a proinflammatory cascade that likely contributes to neuroinflammation.

## Methods

### Cells and viruses

The primary isolate (termed SBCMV) was provided by Dr. Ravit Arav-Boger, Johns Hopkins University, and obtained from the urine of a congenitally infected infant with disseminated HCMV disease. Institutional review board (IRB) exemption for the use of this isolate was given by Johns Hopkins Hospital. Primary human brain vascular pericytes and astrocytes were obtained from Cell Systems (Kirkland, WA, USA). Primary BMVEC cells were a kind gift from Dr. Milan Fiala (UCLA, Los Angeles, CA, USA) [25]. All cells were maintained at low passage in complete pericyte medium from ScienCell Corporation (Carlsbad, CA, USA). Cells were trypsinized and plated in uncoated 100 cm^2^ dishes or uncoated 4.2 cm^2^/well glass chamber slides at a density 1 × 10^6^ and 2.5 × 10^5^ cells per dish and well, respectively. Heat-killed SBCMV was prepared by heating the viral inoculum to 65°C for 30 minutes in a water bath [26].

### Microarray analysis

The GeneChip Human Genome U133 Plus 2.0 array (Affymetrix, Santa Clara, CA, USA) with complete coverage of the human genome containing over 47,000 transcripts was used to identify gene expression changes in primary human brain vascular pericytes three days postinfection. Several proinflammatory cytokines that were upregulated after SBCMV infection were selected for further study. A subgroup of genes identified as having the highest fold transcription levels associated with neuroinflammation were selected for qRT-PCR analysis.

### Cytomegalovirus infection of pericytes and RNA isolation

Pericytes were infected with SBCMV at a multiplicity of infection (MOI) of 0.1 and virus adsorption was allowed for three hours, then the inoculum was removed and replaced with fresh media. Uninfected pericytes were used as mock-infected controls. Cultures were examined daily for evidence of cytopathology and at three days postinfection, cells were harvested, pelleted, and total RNA extracted at a density of 3 × 10^6^ cells using an RNeasy Mini Kit (Qiagen, Valencia, CA, USA). Extracted RNA was treated with 0.4 units/ml of RNase-free DNase (Qiagen, Valencia, CA, USA). RNA purity was first determined by spectrophotometric analysis, then resuspended in RNase-free water at a concentration of 200 ng/μl and stored at −80°C. RNA quality was assessed using an Agilent 2100 bioanalyzer (Agilent Technologies, Santa Clara, CA, USA).

### Electron microscopy

Three days postinfection, pericytes were washed twice in sodium cacodylate buffer pH 7.4, fixed in 2.5% glutaraldehyde for one hour and held at 4°C for an additional twenty-four hours. Monolayers were rinsed twice with 0.1 M sodium cacodylate buffer (pH 7.4), fixed in 1% osmium tetraoxide for one hour, washed twice for five minutes in cacodylate buffer and dehydrated in graded ethanol. HCMV-infected pericytes were pelleted for 10 minutes at 1500 rpm in a Sorvall Legend RT centrifuge (ThermoFisher Scientific, Waltham, MA, USA). Pellets were embedded in spur resin, sectioned for standard electron microscopy (EM) analysis and visualized on a Phillips CM12 electron microscope (Phillips Research, Eindhoven, The Netherlands).

### Western blot

Cell extracts were prepared using RIPA lysis buffer [50 mM Tris pH 7.5, 150 mM NaCl, 2 mM ethylenediaminetetraacetic acid (EDTA) pH 8.0, 1% NP40, 0.5% sodium deoxycholate, 0.1% sodium dodecyl sulfate (SDS), and proteinase inhibitor]. Lysates were placed on ice for 30 minutes and then clarified by centrifugation. Total protein was measured by bicinchoninic acid (BCA) assay (Pierce; ThermoFisher Scientific, Waltham, MA, USA). Fifteen μg of protein lysates from paired, mock and infected samples were fractionated in 4 to 20% SDS-PAGE gels, transferred to nitrocellulose membranes, blocked with 5% milk, 0.1% TBST (0.1% Tween 20, 20 mM Tris, 150 mM NaCl) and incubated at 4°C overnight with a monoclonal antibody to either major immediate early gene (MIE) (mAb 810 recognizes MIE IE1 and IE2, (Millipore, Temecula, CA, USA) or to human cytomegalovirus phosphorylated envelope protein expressed at late times during virus replication (pp65) (Vector Laboratories, Burlingame, CA, USA), both at a 1:2000 dilution. Membranes were washed five times in 0.1% TBST and incubated for one hour followed by incubation with a secondary antibody donkey anti-mouse peroxidase conjugate (Santa Cruz Biotech, Santa Cruz, CA, USA) at a dilution of 1:10,000. Immunoreactive bands were detected with SuperSignal West Dura Extended Substrate (Pierce; ThermoFisher Scientific, Waltham, MA, USA) following exposure to X-ray film.

### RT-PCR

Total RNA was extracted from SBCMV-infected pericytes and controls using a Qiagen RNeasy Mini Kit (Qiagen, Valencia, CA, USA). RNA was DNase-treated prior to elution on the column according to the manufacturer’s recommendations. Messenger RNA in 1 μg of each sample was primed using oligo-dT and reverse transcribed with a high-capacity cDNA reverse transcription kit (Applied Biosystems, Foster City, CA, USA). Gene-specific primer pairs included C chemokine (C-C motif) ligand 5/Rantes (CCL5/Rantes), chemokine (C-X-C motif) ligand 11/I-TAC (CXCL11/I-TAC), and (CXCL8/IL-8) and 10 to 200 ng of cDNA for RT-PCR amplification, using PuReTaq Ready-To-Go PCR beads (GE Healthcare, Buckinghamshire, UK). PCR was carried out in a MJ Mini thermal cycler (Bio-Rad Laboratories, Hercules, CA, USA) in a final volume of 25 μl. The cycling protocol used was 95°C for five minutes, 55°C for thirty seconds, and 72°C for one minute for thirty-six cycles, with a final extension at 72°C for ten minutes. PCR products were electrophoresed in 1.5% agarose and DNA bands visualized by ethidium bromide. Primers for RT-PCR amplification were designated as: HCMV MIE 1 forward 5′-CCAAGCGGCCTCTGATAACCAAGCC-3′, reverse 5′-CAGCACCATCCTCCTCTTCCTCTGG3′ (exon 4, 435 bp); radical S-adenosyl methionine domain-containing protein 2 (RSAD2) forward 5′-CTTTGTGCTGCCCCTTGAGGAA- 3′, reverse 5′CTCTCCCGGATCAGGCTTCCA3′ (256 bp); CXCL8/IL-8 forward 5′-TGGGTGCAGAGGGTTGTG-3′, reverse 5′-CAGACTAGGGTTGCCAGATTTA-3′ (532 bp); CXCL11/I-TAC forward 5′- TTAAACAAACATGAGTGTGAAGGG-3′, reverse 5′- CGTTGTCCTTTATTTTCTTTCAGG-3 (228 bp); and CCL5/Rantes forward 5′- GGCAGCCCTCGCTGTCATCCTCA-3′, reverse 5′ –CTTGATGTGGGCACGGGGCAGTG-3′. Gyceralaldehyde phosphate dehydrogenase (GAPDH) forward 5′- TGATGACATCAAGAAGGTGGTGAA-3′, reverse 5′-TCCTTGGAGGCCATGTGGGCCAT-3′ (256 bp) was amplified in mock and infected cells as a loading and quality control.

### Real-time qPCR

Real-time PCR was performed in 96 well optical plates (Sorenson Bioscience, Inc., Salt Lake City, UT, USA) with cDNA using the MyiQ Single-Color Real-Time PCR Detection System (Bio-Rad Laboratories, Hercules, CA, USA) in 25 μl reaction volumes. A master mix was made according to manufacturer’s instructions using SYBR green supermix (Bio-Rad Laboratories, Hercules, CA, USA) or VeriQuest master mix (Affymetrix, Santa Clara, CA, USA) for amplification of high GC-content cDNAs. Forward and reverse primers were used at a concentration of 250 nM per well, made in RNase/DNase-free H_2_O. Primer sequences for qPCR were as follows: TNF-alpha forward 5′- CAGAGGGAAGAGTTCCCCAG -3′, reverse 5′-CCTTGGTCTGGTAGGAGACG- 3′; IL-1beta forward 5′-CAAATTCGGTACATCCTCGAC-3′, reverse 5′- GTCAGGGGTGGTTATTGCATC-3′); and IL-6 forward 5′- AAACAGATGAAGTGCTCCTTCCAGG-3′, reverse 5-TGGAGAACACCACTTGTTGCTCCA- 3′. The cDNAs from mock-infected and SBCMV-infected pericytes were diluted 1:3 using RNase/DNase-free H_2_O; 3 μl of this dilution was added to each well, and control wells substituted water for cDNA. The cycling sequence included 95°C for three minutes, 95°C for fifteen seconds, 60°C for one minute, 95°C for one minute, 55°C for one minute, and 55°C for thirty seconds for eighty-one total cycles. A GAPDH primer set was included for normalization. Data analysis was performed using Bio-Rad iQ5 optical system software version 2.0.

### Immunofluorescence

Chamber slides containing either infected or uninfected cells were washed twice with phosphate buffer saline (PBS) pH 7.4, air dried, and fixed in absolute methanol for 10 minutes. Cells were air dried for fifteen minutes, hydrated in Tris saline (pH 7.4) for five minutes, and then incubated for one hour with monoclonal antibodies to the HCMV MIE at a 1:50 dilution (MIE, mAb 810, Millipore, Temecula, CA, USA) or the HCMV tegument protein pp65 (UL83) at 1:50 (Vector Laboratories, Burlingame, CA, USA). Dual-labeled immunofluorescence was performed using a mouse monoclonal to HCMV MIE and goat polyclonal antibodies at 1:100 dilution to human CXCL8/IL-8, CXCL11/I-TAC, and CCL5/Rantes purchased from R&D Systems (Minneapolis, MN, USA). Glial fibrillary acid protein (GFAP), (Millipore, Temecula, CA, USA), von Willebrand factor (VWF) (Millipore, Temecula, CA, USA) and neuron*-*glial antigen 2 (NG2) proteogylcan (Santa Cruz Biotech, Santa Cruz, CA, USA) were used at a 1:50 dilution for immunofluorescent staining of astrocytes, brain microvascular endothelial cells (BMVEC) and pericytes, respectively. Cells were washed three times with Tris saline and then incubated at 37°C for thirty minutes with a combination of secondary donkey anti-mouse Immunoglobulin G (IgG) antibodies conjugated with rhodamine isothiocyanate (RITC), and donkey anti-goat antibody conjugated with fluroescein isothiocyanate (FITC), (Jackson ImmunoResearch, West Grove, PA, USA) at a 1:100 dilution in PBS. Cells were washed another three times in Tris saline and mounted with Vectashield mounting media (Burlingame, CA, USA) containing 1.5 μg/ml of 4′,6-diamidino-2-phenylindole (DAPI). Fluorescence was photographed with a Nikon TE 2000 S fluorescent microscope mounted with a charge-coupled device (CCD) camera (Nikon, Tokyo, Japan).

### Human cytokine ELISA

The effects of HCMV exposure on pericyte expression of cytokines was measured with a human angiogenesis cytokine ELISA assay kit, profiling angiogenic cytokines (Signosis Sunnyvale, CA, USA). Mock-infected, pericytes exposed to heat-killed virus and SBCMV-infected pericytes were plated at a density of 5 x 10^5^ cells in 10 cm dishes and grown to 90% confluence. Cell supernatants were then assayed for TNF-alpha, IL-6 and tumor growth factor-beta (TGF-beta), according to the manufacturer’s protocol. Quantitative analysis of IL-6 and TNF-alpha secreted from SBCMV-infected pericyte supernatants after 24 hours were monitored using human TNF-alpha and human IL-6 cytokine ELISA assays from Signosis according to the manufacturer’s protocol.

### Immunohistochemistry

Dual-labeled immunohistochemistry (IHC) was performed as previously described [27] Archival brain tissue from an HIV-infected patient was obtained from the Texas Repository for AIDS Neuropathogenesis Research (Galveston, TX, USA). Specimen use was HIPPA compliant and approved by the Meharry Medical College IRB. Brain tissue was histologically identified as positive for HIV and HCMV. Tissue was mounted on chemate slides, paraffin embedded and dual stained by IHC for HCMV MIE in combination with the pericyte marker NG2 proteoglycan (Abcam, Cambridge, MA, USA) [28].

### Statistical analysis

Experiments presented in this study were performed in triplicate (CMV-exposed brain pericytes, astrocytes and BMVEC cell pellets were used for qRT-PCR, while parallel supernatants were pooled for the ELISA assay). Quantitative real-time PCR experiments were replicated three times and normalized to GAPDH.

## Results

### Cytomegalovirus infection of pericytes

**Figure 1:**
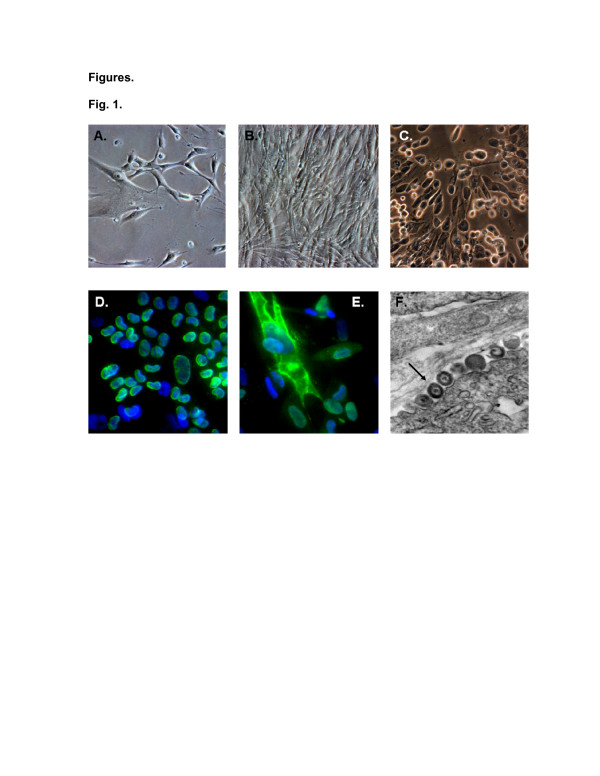
**Primary brain vascular pericytes.** Phase contrast images of: **(A)** an uninfected subconfluent monolayer of primary brain vascular pericytes, **(B)** a confluent monolayer of brain vascular pericytes, and **(C)** pericytes 72 hours after infection with SBCMV. Immunofluorescence staining of SBCMV-infected pericytes for **(D)** HCMV MIE protein and **(E)** pp65 late protein. **(F)** TEM of SBCMV-infected pericytes showing HCMV virions in the cytoplasm (see arrow). With the exception of the TEM, images were taken on a Nikon TE2000S microscope (200x magnification). HCMV, human cytomegalovirus; MIE, major immediate early protein; SBCMV, primary HCMV isolate from a patient; TEM, transmission electron microscopy.

Primary brain vascular pericytes exhibited characteristic long extensions of cytoplasm in subconfluent cultures (Figure 1A), appeared fibroblastic when confluent (Figure 1B), and had typical cytomegalic cytopathology three days postinfection with the SBCMV clinical isolate (Figure 1C). Pericytes supported full lytic replication of SBCMV, as more than 80% of cells were infected as demonstrated by the production of HCMV MIE 1 and 2 and the viral tegument protein pp65 (Figure 1D, E). These results were performed in triplicate using the same viral inoculum. Transmission electron microscopy (TEM) analysis revealed typical cytomegalovirus virions in SBCMV-infected pericytes three days postinfection (Figure 1F).

### Cytomegalovirus protein expression in pericytes and upregulation of proinflammatory cytokine proteins and mRNA

**Figure 2:**
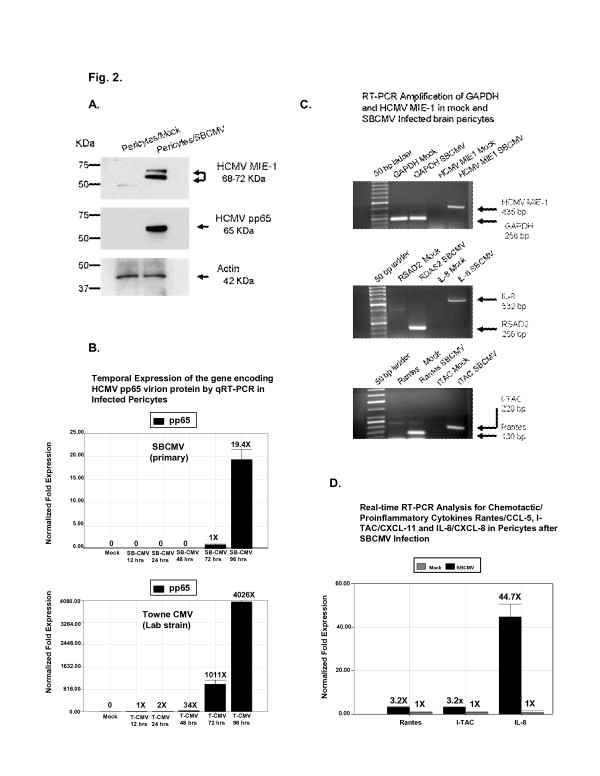
**Protein expression in mock- and SBCMV-infected brain pericytes. (A)** Western blot showing expression of HCMV MIE and pp65 virion proteins in pericytes infected with SBCMV for 72 hours. Actin was the loading control. **(B)** Temporal expression of HCMV pp65 virion protein by real-time PCR in infected pericytes (SBCMV or Towne CMV) at 12, 24, 48, 72, and 96 hours postinfection. **(C)** Semi-quantitative RT-PCR for HCMV MIE, GAPDH, CXCL8/IL-8, RSAD2, CXCL11/I-TAC and CCL5/Rantes (72-hour exposure). GAPDH was the loading control. **(D)** Real time RT-PCR analysis for CCL5/Rantes, CXCL11/I-TAC, and CXCL8/IL-8 in pericytes 72 hours after SBCMV infection. This experiment was replicated three times, and amplifications were performed in triplicate and normalized to GAPDH. CCL5/Rantes, chemokine (C-C motif) ligand 5/Rantes; CXCL8/IL-8, blood–brain barrier; CXCL11/I-TAC, chemokine (C-X-C motif) ligand 11/I-TAC; GAPDH, gyceralaldehyde phosphate dehydrogenase; HCVM, human cytomegalovirus; MIE, major immediate early protein: pp65, human cytomegalovirus phosphorylated envelop protein expressed at late times during virus replication; RSAD2, radical S-adenosyl methionine domain-containing protein 2; RT-PCR, reverse transcription polymerase chain reaction; SBCMV, primary HCMV isolate from a patient.

Using Western blots, we observed HCMV MIE 1, 2 and the pp65 late virion protein expression in pericytes infected with SBCMV compared to mock-infected control cells (Figure 2A). Temporal expression of the gene encoding HCMV pp65 virion protein was determined by qRT-PCR in pericytes infected with either the SBCMV isolate or the Towne lab HCMV strain after 12, 24, 48, 72, or 96 hours of incubation. With the SBCMV clinical isolate, we observed marginal expression of pp65 mRNA in infected pericytes beginning at 72 hours and a 19.4-fold increase in pp65 mRNA at 96 hours postinfection compared to mock-infected controls (Figure 2B). With the Towne laboratory strain of HCMV we observed a 34-fold, 1,011-fold and a 4,026-fold increase in pp65 mRNA in infected pericytes at 48, 72, and 96 hours respectively (Figure 2B). By semi-quantitative real-time PCR we observed upregulation of IL-8, CXCL11/I-TAC, and CCL5/Rantes in SBCMV-infected pericytes compared to mock-infected control cells (Figure 2C). Transcriptional analysis by semi-quantitative RT-PCR revealed HCMV MIE 1 transcripts expressed in infected cells but not controls (Figure 2C). Using RT-PCR amplifications for proinflammatory cytokines CXCL8/IL-8, CXCL11/I-TAC, and CCL5/Rantes, we observed increased mRNA expression for all three cytokines in SBCMV-infected pericytes (Figure 2C). RSAD2 [29,30] was amplified as a control for genes specifically upregulated after HCMV infection (Figure 2B). Quantitative real-time PCR analysis indicated that CCL5/Rantes, CXCL11/I-TAC, CXCL8/IL-8 were upregulated after SBCMV infection by a factor of 3.2-fold, 3.2-fold and 44.7-fold, respectively, when normalized to transcription levels in mock-infected cells [31-33] (Figure 2D).

### Cellular upregulation of proinflammatory cytokines in SBCMV-infected brain pericytes

**Figure 3:**
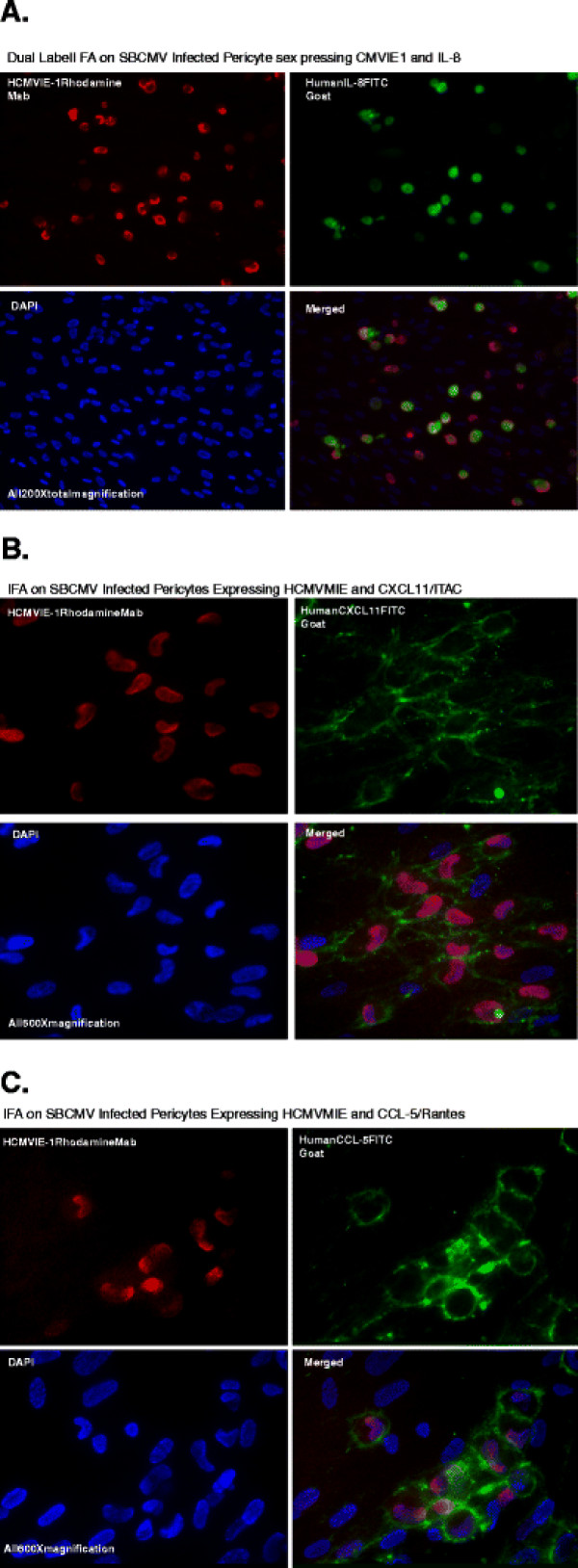
**Dual-labeled immunofluorescent staining of SBCMV-infected pericytes showing colocalization (merged images) of proteins. (A)** Infected pericytes expressing MIE proteins and CXCL8/IL-8. **(B)** Infected pericytes expressing MIE proteins and CXCL11/I-TAC. **(C)** Infected pericytes expressing MIE proteins and CCL5/Rantes. CCL5/Rantes, chemokine (C-C motif) ligand 5/Rantes; CXCL8/IL-8, blood–brain barrier; CXCL11/I-TAC, chemokine (C-X-C motif) ligand 11/I-TAC; MIE. major immediate early protein; SBCMV, primary HCMV isolate from a patient.

**Figure 4:**
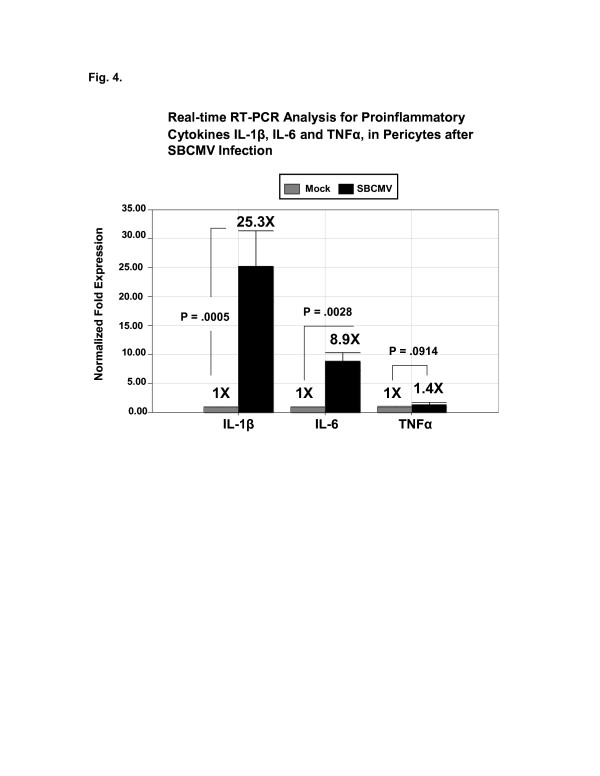
**Real time RT-PCR analysis for proinflammatory cytokines.** Cytokine IL-1 beta, IL-6 and TNF-alpha expression in pericytes 72 hours after SBCMV infection. This experiment was replicated three times, and amplifications were performed in triplicate and normalized to GAPDH. GAPDH, gyceralaldehyde phosphate dehydrogenase; RT-PCR, reverse transcription polymerase chain reaction; SBCMV, primary HCMV isolate from a patient.

After performing dual-labeled immunofluorescence for the HCMV MIE nuclear antigen (RITC-labeled) and the respective chemokine (FITC-labeled), we observed an increase in cellular expression of IL-8, CXCL11, and Rantes FITC, stained with donkey anti-goat antibodies (Figure 3A, B, C). IL-1 beta (25-fold) and IL-6 (8-fold) were also upregulated in SBCMV-infected pericytes by qRT-PCR when compared to mock-infected control cells (Figure 4). We observed statistical significance for IL-1 beta and IL-6 but not TNF-alpha transcription.

### Pericytes are more permissive for SBCMV infection than astrocytes and brain microvascular endothelial cells

**Figure 5:**
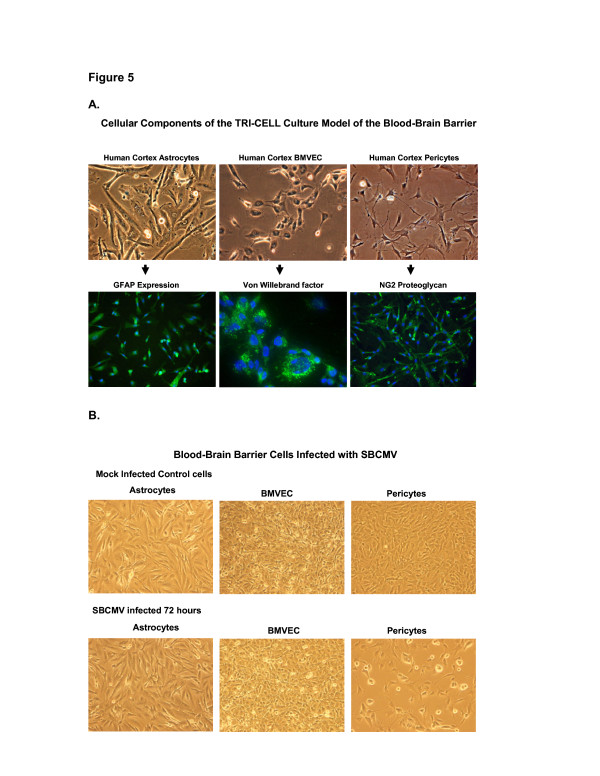
**CMV infection of blood–brain barrier cellular components.** Human brain cortical astrocytes, BMVEC and pericytes representing the major cellular components of the blood–brain barrier were infected with SBCMV. Cells cultivated to 50% confluence were stained with representative markers. **(A)** Astrocytes were stained for glial fibillary acidic protein (GFAP), BMVEC for von Willebrand factor (vWF) and brain pericytes with NG2 proteoglycan antibodies. **(B)** All three cell types were infected with SBCMV for 72 hours. BMVEC, brain microvascular endothelial cells; GFAP, glial fibrillary acidic protein; NG2, neuron*-*glial antigen 2; SBCMV, primary HCMV isolate from a patient; vWF, von Willebrand factor.

**Figure 6:**
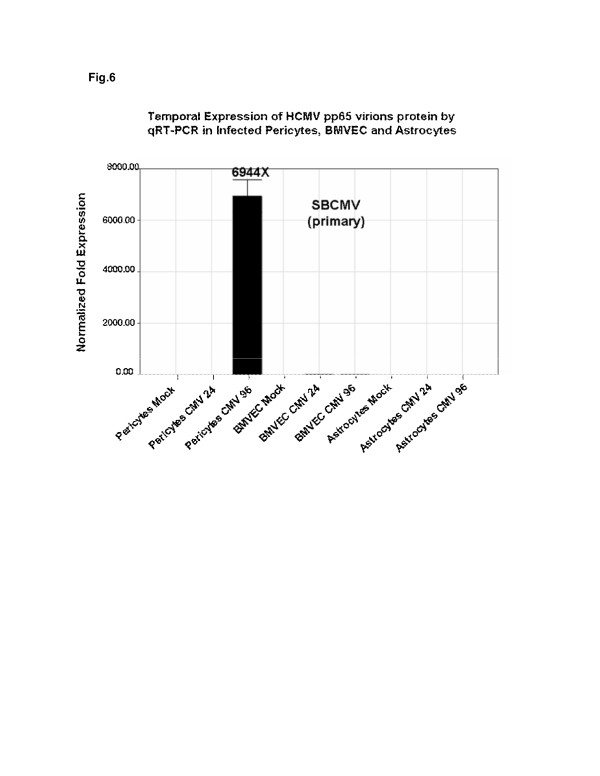
**Expression of SBCMV pp65 virion protein mRNA by pericytes, BMVEC and astrocytes.** Real-time PCR analysis of pp65 late virion protein mRNA levels in SBCMV-infected pericytes, BMVEC and astrocytes. cDNA was prepared from total RNA isolated at 24 and 96 hours postinfection along with RNA from mock-infected control cells. Black bars indicate fold transcription for SBCMV-infected pericytes, BMVEC and astrocytes. This experiment was replicated three times and normalized to GAPDH. BMVEC, brain microvascular endothelial cells; GAPDH,gyceralaldehyde phosphate dehydrogenase; pp65, human cytomegalovirus phosphorylated envelop protein expressed at late times during virus replication; PCR, polymerase chain reaction; SBCMV, primary HCMV isolate from a patient.

**Figure 7:**
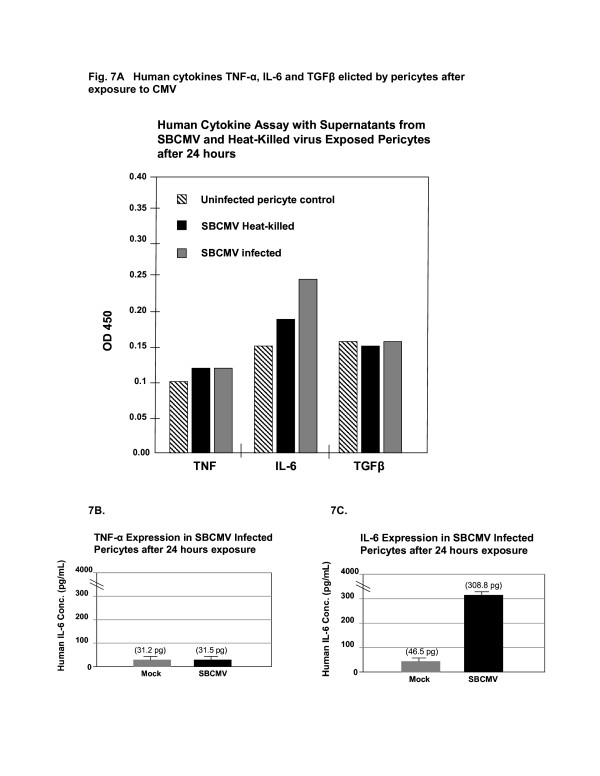
**Human cytokine ELISA assay.** Analysis of human cytokines after SBCMV infection of brain vascular pericytes. A human cytokine ELISA assay was performed on supernatants from SBCMV-infected pericytes, SBCMV heat-killed pericytes and mock-infected cells. Shown are the relative amounts of TNF-alpha, IL-6 and TGF-beta in cell culture supernatants elicited 24 hours postexposure **(A)**. Quantitative measurements IL-6 and TNF-alpha secreted from pericytes in infected cell supernatants 24 hours postinfection was based on optical density utilizing a standard curve for human IL-6 **(B)** and human TNF-alpha **(C)**. Analysis was performed in triplicate and values were expressed in picograms/milliliter. IL, interleukin; SBCMV, primary HCMV isolate from a patient; TGF-beta, tumor growth factor-beta; TNF-alpha, tumor necrosis factor-alpha.

Cultured human cortical astrocytes, BMVEC and pericytes were stained positive for representative markers glial fibrillary acidic protein (GFAP), von Willebrand factor (vWF) and NG2 proteoglycan, respectively (Figure 5A). All three cell types were separately exposed to SBCMV for 72 hours (Figure 5B). Only pericytes cultures developed cytomegalic cytopathology compared to astrocytes and BMVEC (Figure 5B). Astrocytes and BMVEC cells exposed to SBCMV appeared no different than mock-infected cells, in that there was no evidence of infection by microscopy (Figure 5B). We then wanted to examine for evidence of viral lytic gene expression using qPCR. mRNA expression for the CMV late virion protein pp65 revealed that pericytes expressed pp65 mRNA at a very high level compared to controls, with no detection above background expression for astrocytes and BMVEC cells (Figure 6). We then wanted to examine the expression of proinflammatory cytokines elicited by pericytes after SBCMV exposure. Utilizing a human cytokine ELISA assay with supernatants from SBCMV and heat-killed virus exposed pericytes 24 hours after exposure, we observed a marginal increase in TNF-alpha in pericytes exposed to both SBCMV and heat-killed virus (Figure 7A). In addition, we also showed an increase in IL-6 in pericytes exposed to both SBCMV and heat-killed virus, although a higher level of IL-6 was observed in those pericytes exposed to untreated SBCMV compared to heat-killed virus. No significant change in TGF-beta levels was observed in 24-hour virus-exposed pericytes when compared to uninfected control cells (Figure 7A). Analysis of TNF-alpha and IL-6 secreted from pericytes in infected cell supernatants showed no induction of TNF-alpha compared to mock-infected controls (Figure 7B) and a 6.6-fold increase in IL-6 protein levels was observed in SBCMV-infected cell supernatants when compared to controls levels (Figure 7C).

### Cytomegalovirus infects pericytes *in vivo*

**Figure 8:**
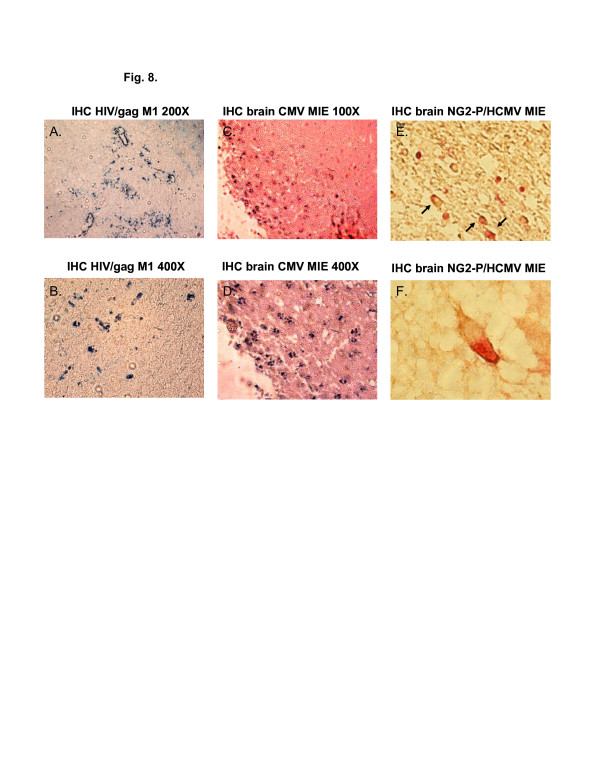
**Immunohistochemical staining of HIV and CMV coinfected brain tissue.** IHC staining of archival human hippocampus brain tissue coinfected with HCMV from an HIV-infected patient. **(A/B)** Brain tissue IHC-stained for HIV Gag) using True Blue (Kirkegaard & Perry Laboratories, Gaithersburg, MD, USA) as a substrate for immunoperoxidase. Photographs in A and B were taken at 200x and 400x magnification, respectively. **(D/C)** IHC-stained brain tissue for the HCMV MIE gene protein using True Blue as an immunoperoxidase substrate. Photographs were at 200x and 400x magnification. **(E)** Brain tissue IHC dual-stained for the pericyte marker NG2 proteogylcan using 3,3-diaminobenzidine (DAB) staining cells brown and CMV MIE gene protein using Vector Red (Vector Laboratories, Burlingame, CA, USA) as a substrate for alkaline phosphatase (dual-stained indicated by arrows). This photograph was taken at 200x magnification. **(F)** Brain tissue dual-stained by IHC for NG2 proteoglycan and HCMV MIE gene protein showing a cell with a brown-colored cytoplasm and a red nucleus. This image was taken at 600x magnification. All images were taken with a Nikon TE2000S microscope fitted with a CCD camera (Nikon, Tokyo, Japan). CMV, cytomegalovirus; DAB, 3,3-diaminobenzidine; IHC, immunohistochemistry; HCMV, human cytomegalovirus; MIE, major immediate early protein; NG2, neuron*-*glial antigen 2.

Single and dual IHC staining was performed on archival brain tissue from an HIV-infected patient with HCMV-disseminated CNS disease. We demonstrated the presence of HIV by single label IHC (Figure 8A, B) using HIV group-specific antigen (Gag) antibodies, and for HCMV in (Figure 8C, D) using virus major immediate early gene (MIE) antibodies. To demonstrate CMV infection of pericytes in archival brain tissue, we performed dual-labeled IHC using antibodies for both NG2 proteoglycan, a cytoplasmic marker for pericytes [27, 28], and for HCMV MIE, a nuclear marker to detect CMV-infected pericytes. We demonstrated dual cytoplasmic (NG2 proteoglycan, brown) and nuclear staining (HCMV MIE, red) in CMV-infected archival brain tissue. These results support our *in vitro* proof of concept data indicating that human brain vascular pericytes support CMV infection.

## Discussion

The role of brain vascular pericytes regarding CMV infectivity and virus dissemination into the CNS is unknown. The role of pericytes in HCMV-induced neuroinflammation is also unknown. Determining the mechanisms involved in the dissemination of CMV in the CNS is critical for understanding how CMV infection *in utero* may cause neurodevelopmental abnormalities in children [34,35]. In this study, we found that pericytes are highly permissive for HCMV lytic replication using a low-passaged clinical isolate of human CMV (SBCMV). We observed typical CMV cytopathology and staining patterns for MIE 1, 2 and the late viral protein pp65 as well as virion production consistent with lytic replication in permissive cells [4].

We felt that it was essential to use a low-passaged clinical isolate of HCMV in these studies because laboratory-adapted strains of HCMV are passaged in culture and accumulate deletions as well as genome rearrangements for growth adaptation in fibroblasts, which are routinely used to cultivate CMV in the laboratory [36-38]. Therefore, when we observed a lower level of viral replication kinetics in pericytes for the SBCMV isolate compared to the Towne lab CMV strain, we attributed this finding to the lack of adaptation of a virus that was only passaged in culture twice after initial isolation (Figure 2B). Studies by Cha *et al*. in 1996 [36] showed that human CMV clinical isolates carry up to 19 genes not found in the highly passaged laboratory strains of HCMV. These genome differences may account for the differences in virulence and tissue tropism [39,40].

The loss of HCMV UL128-to-UL150 loci, and specifically the UL128, UL130 and UL131 gene cluster observed in the laboratory-adapted strains of HCMV like AD169 and Towne that are important for virus infection and spread in epithelial and endothelial cells, due to extensive passage in fibroblasts, appears not to be required for infection and virus spread in pericytes infected with HCMV Towne [41].

A comparative analysis of SBCMV infectivity *in vitro* with other cellular components of the BBB (astrocytes and BMVEC) suggests that pericytes are the most permissive of this group for CMV lytic replication. Thus they may serve as a viral CNS amplification reservoir that could greatly enhance CMV dissemination into the brain. In addition, a proinflammatory cytokine cascade was expressed by brain pericytes following SBCMV infection for 72 hours. This included upregulation of chemotactic cytokines CCL5/Rantes, I-TAC/CXCL11, and IL-8/CXCL8, as well as IL1-beta and IL-6 with no induction of TNF-alpha (Figure 4) which correlates with our semi-quantitative ELISA assay (Figure 7A). These cytokines are mainly chemotactic for neutrophils, T cells, basophils and eosinophils and would serve to contribute to neuroinflammation at the blood–brain barrier (Table 1).

**Table 1:** Microarray results three days after SBCMV infection of brain vascular pericytes representing the highest fold change in transcription linked to neuroinflammation.

Genes upregulated	Fold change	Some associated functions
RSAD2	390	Protein directly induced by cytomegalovirus
ISG20	380	Interferon-stimulated exonuclease
CCL5	364	Chemotactic cytokine for activated T cells, eosinophils and basophils
CXCL11	337	Chemotactic cytokine for activated T cells
CCL5	141	Chemotactic cytokine for activated T cells, eosinophils and basophils
IFI27	117	Interferon alpha inducible gene/inflammation
TAC1	111	Neurotransmitter, induction of behavioral responses
CCL20	76	Chemotactic cytokine for lymphocytes and neutrophils
HERC5	60	Interferon-induced ubiquitin-E3 protein ligase
IL8	4	Chemotactic cytokine for neutrophils

CCL5, C chemokine (C-C motif) ligand 5; CCL20, chemokine (C-C motif) ligand 20; CXCL11, chemokine (C-X-C motif) ligand 11; HERC5, probable E3 ubiquitin-protein ligase HERC5; IFI27, interferon alpha-inducible protein 2; IL8, interleukin 8; ISG20, interferon-stimulated gene 20 kDa protein; RSAD2, radical S-adenosyl methionine domain-containing protein 2; TAC1, tachykinin precursor 1.

Studies that demonstrate CMV infection of astrocytes report the production of chemokines, mainly CCL2 and cytokine transforming growth factor beta (TGF-beta) [42,43]. In these studies, none of the proinflammatory cytokines used (TNF-alpha, IL-1beta, and IL-6) were found to be induced in astrocytes in a mouse model system using murine cytomegalovirus (MCMV) [42]. Using a low-passaged clinical isolate of HCMV (SBCMV) to infect a commercial source of primary human astrocytes at a low multiplicity of infection, we found no clear evidence of CMV infection in these cells after 72 hours in culture and by qPCR amplification (Figures 6 and 7).

There are *in vitro* reports (using laboratory-adapted strains of CMV in human, murine and nonhuman primate model systems) that CMV infects both BMVEC and astrocytes [42-45]. However our study, using a low multiplicity of infection with a primary clinical HCMV isolate obtained from a child with disseminated CMV disease, found no evidence of CMV pp65 message above background in infected BMVEC or astrocytes for up to 96 hours after infection when compared to pericytes (Figure 6). The Towne strain of HCMV has been highly passaged in culture and has accumulated multiple mutations and deletion and is expected to have broader tropism when compared to a low-passaged primary clinical CMV isolate. The HCMV infections we performed were at a low MOI and we monitored cytopathic effects after 72 hours in Figure 5 and, using mRNA from the same cultures, we looked for pp65 late transcripts at 24 and 96 hours postinfection, which allows time for productive infection (late gene expression). The infection was performed at low MOI using a primary HCMV isolate, a 24- and 96-hour time point postinfection, and amplification of the late protein pp65 transcripts likely contributed to the low level of productive infection seen in astrocytes. We observe that longer time periods postinfection, a higher MOI, and the use of a laboratory-adapted strain of virus will produce higher levels of productive infection in fibroblasts and pericytes. Even more, we observed that SBCMV-infected pericytes elicited higher levels of IL-6 when compared to mock-infected or heat-killed SBCMV controls. Marginal increases in TNF-alpha levels were observed in pericyte cultures exposed to both SBCMV and heat-killed virus for 24 hours (Figure 7A), while no change was observed in TGF-beta levels in virus-exposed cells compared to uninfected control cells over this time period. Increased levels of IL-6 and no induction of TNF-alpha were observed in SBCMV-infected pericyte supernatants at 24 hours postinfection. As we were not able to obtain archival brain tissue from newborns that were only CMV infected, we utilized CMV-infected brain tissue from a patient coinfected with HIV. Because CMV is a latent opportunistic pathogen that undergoes reactivation from latency in individuals that are immune-compromised, HIV-infected patients can develop CMV neuropathology [46-49]. Using HIV-CMV-infected archival human brain tissue, we demonstrated CMV infection of brain pericytes using dual-labeled IHC. We observed both HIV-positive cells in this hippocampal tissue as well as CMV cytomegalic cells with a characteristic CMV MIE nuclear staining pattern. In addition, we demonstrated brain cells staining positive for the pericyte marker NG2 proteoglycan, which co-stained for HCMV MIE gene protein, suggesting that pericytes support CMV infection *in vivo* (Figure 8). We realize that no specific pericyte marker has been identified. The use of alpha smooth muscle actin (alpha-SMA) has been employed by investigators to identify pericytes *in situ*, however in the brain, only pericytes located near arterioles routinely stain positive for alpha-SMA. In primary cultures, only 5% of newly isolated capillary pericytes stain positive for this marker [22]. Studies by Ozerdem *et al*. [28] suggested that NG2 proteogylcan is expressed exclusively by pericytes (mural cells) during vascular morphogenesis. With this reasoning we used NG2 proteogylcan as a marker for pericytes in our IHC experiments.

## Conclusion

**Figure 9:**
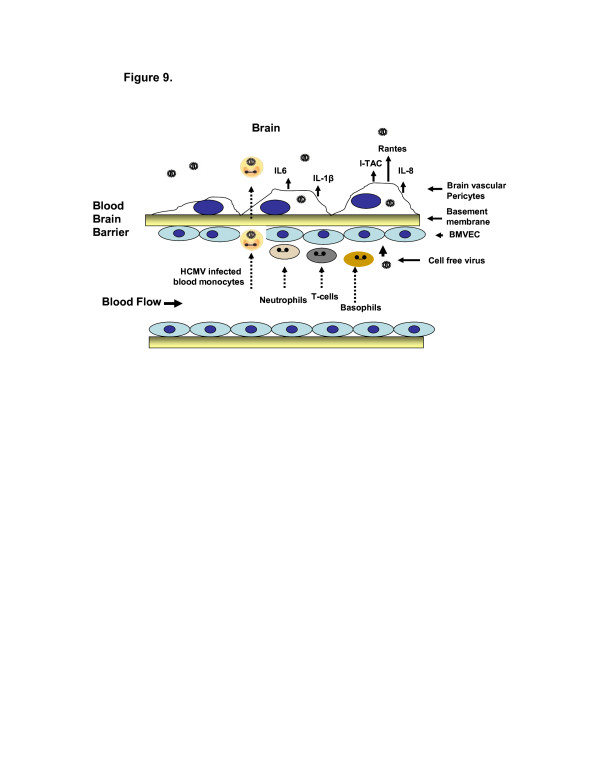
**Model of HCMV dissemination across the blood–brain barrier including pericytes and a CMV-elicited proinflammatory cytokine cascade.** A working model of HCMV dissemination across the blood–brain barrier involving pericytes and a proinflammatory cytokine cascade. (H)CMV, (human) cytomegalovirus.

Based in part on these findings, we proposed a model of HCMV dissemination from the blood across the BBB into the brain (Figure 9). As HCMV traffics into the brain, the virus encounters brain microvascular endothelial cells that may be marginally permissive for infection [50,51]. HCMV-infected blood monocytes may also gain access to the brain via chemotactic factors like monocyte chemotactic protein 1 (MCP-1) [52]. Cell-free virus could also gain access to the brain via pinocytosis or paracellular transport between brain microvascular endothelial cells (Figure 9). All routes require HCMV to interact with pericytes. Therefore we propose that brain vascular pericytes serve as a virus amplification reservoir at the BBB interface and, upon infection, they express a proinflammatory cytokine cascade that could result in increased inflammation and contribute to CMV neuropathology. HCMV upregulation of proinflammatory cytokines IL-1beta, IL-6 and TNF-alpha in permissive cell has been extensively studied. At early times postinfection, several studies have shown HCMV activation of IL-1, TNF-alpha, nuclear factor-kappa B (NF-kB) and various mitogen activated protein kinases (MAPKs) [53-57]. A second phase of activation occurs after virus entry, induced partly by the major viral transcriptional activators IE1 and IE2, which are thought to be required for initial virus replication. Stimulation of IL-1 and TNF-alpha pathways may also support viral dissemination via recruitment of permissive cells, as a result of increased chemokine secretion such as IL-8, IL-6, and Rantes [58-61]. More recent studies are beginning to suggest the ability of HCMV to suppress IL-1 and TNF-alpha signaling pathways at different times after infection [62,63]. However, the exact mechanisms involved in HCMV regulation of proinflammatory signaling pathways will require further investigation. Taken together, these studies suggest that as HCMV traffics the neurovasculature, virus infection of pericytes is imminent, resulting in infection amplification and the induction of cytokines that heightens the inflammatory state of the CNS microenvironment.

The model in Figure 9 indicates how HCMV might interact with pericytes leading to several potential responses. Infected blood monocytes could either extravasate between BMVEC and transmit infection to pericytes via cell-to-cell contact, or cell-free virus could pass between BMVEC via paracellular transport, to directly infect pericytes. In support of this model, we find pericytes to be fully permissive for HCMV infection. We also suggest that pericytes directly serve as amplification sites for productive viral infection, which would allow CMV to disseminate further into the brain parenchyma.

CMV infection of pericytes would result in a cytokine cascade that would serve to attract inflammatory cells such as neutrophils, basophils and T cells that would in aggregate contribute to neuroinflammation. Figure 9 represents a summary of our findings and exemplifies a model of our hypothesis with regard to HCMV interaction with cellular components of the blood–brain barrier. We are aware that a congenital infection occurs during prenatal life when the blood–brain barrier is still very immature and leaky, allowing more access of HCMV to the brain. Still these data are provocative and require investigators to take a closer look at the role of pericytes in this process.

## Abbreviations

Alpha-SMA, alpha smooth muscle actin; interleukin-8, CXCL8/IL8; BBB, blood–brain barrier; BCA, bicinchoninic acid; BMVEC, brain microvascular endothelial cells; CCD, charge-coupled device camera; CCL5/Rantes, chemokine (C-C motif) ligand 5/Rantes; CCL20, chemokine (C-C motif) ligand 20; cDNA, DNA copy of mRNA after reverse transcription; CMV, cytomegalovirus; CNS, central nervous system; CXCL11/I-TAC, chemokine (C-X-C motif) ligand 11/I-TAC; DAB, 3,3-diaminobenzidine; DAPI, 4′,6-diamidino-2-phenylindole; EDTA, ethylenediaminetetraacetic acid; ELISA, enzyme-linked immunosorbent assay; EM, electron microscopy; FITC, fluorescein isothiocyanate; GAPDH, gyceralaldehyde phosphate dehydrogenase; GFAP, glial fibrillary acidic protein; HCMV, human cytomegalovirus; HERC5, probable E3 ubiquitin-protein ligase HERC5; IgG, immumoglobulin G; IFI27, interferon alpha-inducible protein 27; IHC, immunohistochemistry; IL, interleukin; ISG20, interferon-stimulated gene 20 kDa protein; mAb810, monoclonal antibody to human cytomegalovirus major immediate early proteins 1 and 2; MAPKs, mitogen activated protein kinases; MCMV, murine cytomegalovirus; MCP-1, monocyte chemotactic protein-1; MIE 1 and 2, human cytomegalovirus major immediate early gene/proteins 1 and 2; MOI, multiplicity of infection; mRNA, messenger ribonucleic acid; NF-kB, nuclear factor-kappa B; NG2, neuron-glial antigen 2; PAGE, polyacrylamide gel electrophoresis; pp65, human cytomegalovirus phosphorylated envelop protein expressed at late times during virus replication; qRT-PCR, quantitative reverse transcription polymerase chain reaction; RITC, rhodamine B isothiocyanate; RSAD2, radical S-adenosyl methionine domain-containing protein 2; RT-PCR, reverse transcription polymerase chain reaction; SBCMV, primary HCMV isolate from a patient; SDS, sodium dodecyl sulfate; TAC1, tachykinin precursor 1; TEM, Transmission electron microscopy; TGF-beta, tumor growth factor-beta; TJ, tight junction; TNF-alpha, tumor necrosis factor-alpha; vWF, von Willebrand factor.

## Competing interests

The authors declare that they have no competing interests.

## Authors’ contributions

DJA conceived and designed the study. DJA, AMC, SMK, WQZ, and HEV performed the experiments. DJA drafted the manuscript. All authors have read and approved the final version of the manuscript.
